# Fixed drug combination in hypertension and hyperlipidaemia in the developing world

**Published:** 2008-06

**Authors:** YK Seedat

**Affiliations:** Nelson R Mandela School of Medicine, Faculty of Health Sciences, University of KwaZulu Natal, Durban

Cardiovascular disease is the leading cause of death worldwide. In 2002, it was estimated to have caused over 15 million deaths, comprising more than a quarter of all deaths that year.^1^ About 7.2 million of these deaths were due to ischaemic heart disease and 5.5 million were due to cerebrovascular disease. By 2020 it is estimated that there will be 25 million deaths from cardiovascular disease annually and that these deaths will comprise 37% of all deaths that year.

There is some evidence that death rates from cardiovascular disease are stabilising in higher-income countries^2^ but there is little evidence that the incidence of events of non-fatal cardiovascular disease is falling. Already more than four-fifths of all cardiovascular deaths occur in developing countries and the majority of the growth in global cardiovascular disease burden over the next 20 years will be in these regions.^3^ Of the anticipated nine million additional deaths each year attributable to cardiovascular disease in 2020, about eight million are expected to occur in low- and middle-income populations.

In many developing countries, the primary problem to be addressed with fixed drug combinations does not concern incomplete treatment, but rather no treatment among patients unknown to the healthcare system. In this setting, the benefits of fixed-dose combinations (FDC) would be derived primarily from simplification of the process by which patients would be identified and provided with treatment.

FDC drugs can be defined as two or more drugs in a single formulation, each drug having independent modes of action, the combination of which are synergistic or complementary in their effect. ‘Free’ combinations can be defined as two or more drugs in separate formulations, usually taken at the same time. Many consider combination therapy in whatever form to be essential for the treatment of hypertension and other cardiovascular conditions, as well as major infectious diseases such as HIV/AIDS, tuberculosis and malaria, and for the prevention of drug resistance.

There is limited empirical evidence to support or refute the main purported advantages of FDC drugs, namely increased patient adherence and amelioration of antimicrobial resistance.^4^ The Food and Drug Administration (US) formalised the combination rule for prescription drug products, and states ‘two or more drugs may be combined in a single dosage form when each component makes a contribution to the claimed effects, and the dosage of each component (amount, frequency, duration) is such that the combination is safe and effective … for a significant patient population requiring such concurrent therapy as defined in the labeling for the drug’.^5^

The presumed advantages of FDCs are the following: (1) simpler dosage schedule improves compliance and therefore improves treatment outcomes; (2) reduction of inadvertent medication errors; (3) prevents and/or slows attainment of antimicrobial resistance by eliminating monotherapy (one drug is never in circulation by itself); (4) synergistic combinations allow each drug to selectively interface with successive steps in bacterial folate mechanisms; (5) reduced drug shortages by simplifying drug handling and therefore lowering the risk of being ‘out of stock’; (6) only one expiry date simplifies dosing (single products have different expiry dates); (7) the procurement, management and handling of drugs is simplified; (8) lower production, packing and shipping costs; and (9) side effects may be reduced by using one drug of the combination for the purpose.^5^

The presumed disadvantages of FDCs are: (1) they are sometimes more expensive than separate tablets, although not invariably so; (2) there are potential quality problems when drugs are combined, especially with rifampicin in FDCs for tuberculosis; this requires bioavailability testing; (3) if a patient is allergic or has a side effect, the FDC must be stopped and replaced by separate tablets, although this issue exists even with single-dose formulations; (4) dosing is inflexible and cannot easily be regulated to a patient’s needs as each patient has unique characteristics such as weight, age, pharmacogenetics, co-morbidity and effect. This criticism does not, however, apply to tuberculosis or HIV/AIDS where FDCs are made with weight-adjusted dosages; (5) incompatible pharmacokinetics causes irrational combinations because of different elimination half-lives of individual components.^5^ In India, DIMS lists more than 100 irrational combinations which are not approved in any developed country. I believe there is a need to sensitise undergraduates on prescribing and pharmaco-economics. There is a lack of pressure from interest groups to maintain regulatory quality and prevent irrational combinations, which include bioavailability of one or more components.^6^

The key advantages of FDC are that all the individual active pharmaceutical ingredients would be available at low costs; and high-quality, safe and efficacious products would be assured by compliance of research and development with the process. However, manufacture and quality of assurance is technically more demanding. Bio-equivalence and stability, pharmacodynamics and pharmacokinetics would have to be assessed.^5^

FDCs may improve patient adherence. However, despite the importance of improving adherence, there are surprisingly few large, reliable treatment trials on the effect of combined medication on adherence. Many of the trials are suspect because all the studies were too small or had inadequate follow-up time, and were likely to miss small or moderate-sized effects. FDCs reduce the complexity of dosing regimens and there is a reduction of side effects with reduced dosage. However, these studies were small and one cannot draw conclusions.^7^ It is interesting that, in the ADVANCE study, which is the largest trial using a fixed drug combination of perindopril 4 mg and indapamide 1.25 mg daily (11 140 patients, of whom 3 300 were Chinese), adherence to therapy occurred in 73% of the active group versus 74% in the placebo group.^8^

The Oxfam report titled ‘Pharma companies deny medicine to millions’ states that big pharmaceutical companies need to change the way they work, so as to reach 85% of the world’s consumers who don’t have proper access to medicines. The report lists the shortcomings of industry, which: (1) had failed to implement a systematic and transparent tiered pricing policy when prices are set for all essential medicines according to people’s ability to pay; (2) continues largely to not channel research and development into diseases that predominantly affect poor people in developing countries; (3) continues to be inflexible in protecting intellectual property, including challenging poor countries in court to stop using legal public health safeguards; and (4) continues to rely heavily on donations to get affordable medicines to people, even though this is unsustainable and sometimes counterproductive.^9^

Affordability of drugs is defined as the number of days’ wages required for the lowest-paid individual to purchase a one-month supply of generic aspirin (100 mg), atenolol (100 mg); angiotensin converting enzyme (ACE) inhibitor, lisinopril (10 mg), and simvastatin (20 mg) daily. The affordability of treatment for the secondary prevention of coronary artery disease on this regime would be 1.6 days in Bangladesh, 5.1 days in Brazil, 18.4 days in Malawi, 6.1 days in Nepal, 5.4 days in Pakistan and 1.5 days in Sri Lanka.

The reasons for the unavailability of drugs are: bureaucratic factors delay licensure and discourage manufacturers from introducing drugs into low-income countries; manufacturer’s prices are important causes of unaffordability; import tariffs; a lack of comparative price data; distribution costs; and markups by distributors, pharmacies and dispensing doctors.

The methods to improve affordability of drugs are: increase efficiency and volume of production of drugs; prescribe lowest effective dose; clarify treatment guidelines so that manufacturers can concentrate on fewer drugs; negotiate with manufacturers; publicise the lowest price; and reduce the credible threat of government action.

Reduced costs with FDCs are obtained by reduced expenditure in the packaging of medications by pharmaceutical companies. Packaging will be required for one medication instead of three or four; storage, handling and distribution costs will be for a single agent; there will be a saving to the health system due to a single prescription and a corresponding single episode of dispensing; low-cost generic formulations can be used.^10^

FDCs could easily be provided through non-physical medical services. Minimal retraining of staff would be required. There is every chance of benefit, and low risk of harm means that occasional inappropriate treatment of individuals without cardiovascular diseases can be tolerated since it is unlikely to do harm. The number of medication errors made by prescribers, dispensers and patients can be reduced through simplification of treatment regimens.

The pharmaceutical industry, academic and public health sectors are advocating the expansion of the concept of the prevention of secondary cardiovascular disease. A proposed FDC for established ischaemic heart disease could consist of aspirin 75 mg, simvastatin 40 mg, lisinopril 10 mg and atenolol 25 mg. For established ischaemic cerebrovascular disease, aspirin 75 mg, lisinopril 20 mg, simvastatin 40 mg and hydrochlorothiazide 12.5 mg daily could be used. All these drugs are available in generic formulation.

There is evidence in the literature of a 20% reduction in relative risk for antiplatelet therapy,^11^ a further 25% reduction for cholesterol reduction^12^ and a 25% reduction for blood pressure lowering^13^ in the secondary prevention of cardiovascular disease. In a large overview of patients treated with low-dose aspirin, there was no fatal extra-cranial bleeding.^14^ If each of these three risk reductions are achieved together, the overall reduction in the relative risk of cardiovascular events would be 55%. The benefits of low-dose aspirin outweigh the risks as long as patients are at high risk of ischaemic events and low risk of haemorrhagic events. In the developing world, it may be advisable to exclude aspirin, particularly in low-income groups in whom there is a high risk of haemorrhagic strokes.

In other patient groups, for secondary prevention of cardiovascular disease, beta-blockers are probably contra-indicated in peripheral vascular disease and atrial fibrillation. In diabetic patients, the use of aspirin for primary prevention of cardiovascular disease is not proven. There is a strong rationale for aggressive lowering of blood pressure and cholesterol levels.

Generic drugs have been legally defined in France as ‘a copy of an original medicinal drug whereby production and marketing are made possible by the expiry of the patent covering the innovator product.’ Regulatory authorities should require that documentation supporting a generic pharmaceutical product meets the following criteria: manufacture and quality control; product characteristics and labeling; and therapeutic equivalence.

For a generic drug, the quality of the active ingredient takes on great importance. Almost 90% of essential drugs contained in the WHO model list are off-patent and available in generic form. Raw materials can also be generic.^14^ In many developing countries, central purchasing depots have been created and rely on a system of open tendering. However, if this allows drugs to be obtained at very low prices, it does not enforce the need for quality products. Further shortcomings to this kind of method include: a number of suppliers in all countries worldwide will respond to open tenders; there is lack of quality-control laboratories in developing countries. Where they are present, they suffer from under-resourcing in staff, material and finances; and a large proportion of drugs on open tender do not have a marketing authorisation in the country of manufacture.^15^

A strategy to reduce cardiovascular disease by more than 80% was published in 2003.^14^ The design reviewed published meta-analyses of randomised trials and cohort studies and a meta-analysis of 15 trials of low-dose (15–125 mg/daily) aspirin. The results of the study showed that a thiazide, a betablocker and an ACE inhibitor, each at half standard dose, folic acid (0.8 mg) and aspirin (75 mg) daily would benefit one-third of the people taking this polypill from age 55 years, gaining an average of 11 years of life free from an ischaemic heart disease or stroke. The polypill would cause symptoms in eight to 15% of people. This study adds that intervening on all four risk factors would reduce heart attacks and strokes by over 80%.

To achieve this large effect in a population requires a combination treatment taken by everyone above a specified age (say 55 years) as well as younger people with a clinical history of occlusive arterial disease. A combination pill containing six active components could be widely used. Each component has been used in medical practice for more than 10 years with substantial evidence of safety and efficacy. The editorial on the article^16^ titled ‘A cure for cardiovascular disease?’ stated that the combination treatment has an enormous potential, especially in developing countries.

What is needed to realise the potential benefits has created widespread debate on the new paradigm. There are technical solutions in developing and manufacturing the pills so that chemical activity is maintained. Explicit regulatory requirements ideally based on balance of benefit and harm rather than fixed-dose polypharmacy are, intrinsically, trials assessing bioavailability, intermediate endpoint effects, tolerability and adherence. While ensuring those in need get access to medications with clear indications, contra-indications and affordable formulations, systems can be put in place to ensure profits would be made on large volumes rather than wide margins.

The polypill holds promise for people with chronic disease. Only 20% of chronic disease occurs in high-income countries – 80% occurs in low- and middle-income countries and these deaths include equal numbers of men and women. High-cost physician models of care for chronic diseases developed in high-income countries are usually completely unsuitable in lower-income countries. Of 10 000 patients sampled in 10 low- and middle-income countries, it was found about 20% of patients with coronary heart disease were not receiving any aspirin and about half the patients on beta-blockers, which are low cost and widely available, were not receiving them.^17^

A global trial of the polypill was begun in 2007. A three- or four-drug polypill has been supported by WHO. The value for such a pill must be ‘clearly demonstrated rather than simply assumed’.^18^

Further longer-term research initiatives or more FDCs are necessary. Greater reduction is needed in cholesterol levels, combining statins with fibrates or ezetimibe. There is good evidence that low-dose combinations of agents can achieve blood pressure reduction at least comparable to those with full doses of single agents but with a lower incidence of side effects. Additional platelet activity may be obtained combining aspirin and clopidopril. Fish oils are of proven value in cardiovascular diseases. There is insufficient data on the value of folic acid.

Several questions exist regarding the future of FDCs. (1) Although there is clearly a public health need for FDCs, what are the clinically desirable combinations? (2) What is the actual evidence to support the rationale of FDCs? (3) Are the legal bottlenecks to increase FDC access more apparent than real? (4) What are the ‘real world’ formulation and quality-assurance issues? (5) Can there be a standardised regulatory requirement for ‘combination’ products? Should synergy be required for combinations or is this too high a hurdle?^5^
